# Reasons to Use and Disclose Use of Complementary Medicine Use – An Insight from Cancer Patients

**DOI:** 10.5539/cco.v2n2p81

**Published:** 2013-10-07

**Authors:** Kristen Arthur, Juan Carlos Belliard, Steven B. Hardin, Kathryn Knecht, Chien-Shing Chen, Susanne Montgomery

**Affiliations:** 1 School of Public Health, Loma Linda University, Loma Linda, CA, USA; 2 School of Medicine, Loma Linda University, Loma Linda, CA, USA; 3 School of Pharmacy, Loma Linda University, Loma Linda, CA, USA

**Keywords:** Complementary and Alternative Medicine (CAM), Complementary Medicine (CM), cancer therapies, disclosure of CAM

## Abstract

Studies have shown a high prevalence (40–83%) of complementary and alternative medicine (CAM) use among cancer patients in the U.S.A cross-sectional, mixed-methods pilot study was completed. This paper focuses on the quantitative analysis conducted on demographic predictors of complementary medicine (CM) use, reasons to use CM, and disclosure to healthcare provider data. Surveys were interview-administered at the Loma Linda University Medical Center Cancer Center. Participants, 18 years or older, were selected from a convenient sample. Eighty-seven percent (87.9%) of participants reported to have used CM as a cancer treatment and most reported to have used it “to help fight the cancer.” Women were eight-times more likely to use prayer. All non-Caucasian and Hispanic participants reported to use CM as a cancer therapy and none reported to use a CM provider. More women (72%) disclosed their CM use than men (53.3%). Different prevalences and predictors exist when differentiating CM modalities, reasons to use CM vary by gender, and disclosure proportions vary by gender.

## 1. Introduction

Complementary and alternative medicine (CAM) is generally defined as a group of diverse medical and health care systems, practices, and products that are not presently considered to be part of conventional medicine (NCCAM, 2010; Harris & Rees, 2000). It is estimated that 38% of all adults in the United States use CAM each year (Barnes, 2008); however, the inclusion of certain CAM items is inconsistent (NCCAM, 2010). Most Americans who use CAM use complementary medicine (CM) as an adjunct to conventional treatments (NCCAM, 2010). Studies have reported that use of CM among patients with a serious illness, such as cancer, is higher than that of the general population and ranges from 40% to 83% (Boon et al., 2000; Buettner et al., 2006; Cassileth, Lusk, Strouse, & Bodenheimer, 1984; DiGianni, Garber, & Winer, 2002; Ernst & Cassileth, 1998; Ganz et al., 2002; Greenlee et al., 2009; Habermann et al., 2009; Henderson & Donatelle, 2004; Lawsin et al., 2007; Lee, Lin, Wrensch, Adler, & Eisenberg, 2000; Lo, Desmond, & Meleth, 2009; J. J. Mao, Farrar, Xie, Bowman, & Armstrong, 2007; J. J. Mao, 2011; Morris, Johnson, Homer, & Walts, 2000; Richardson, Sanders, Palmer, Greisinger, & Singletary, 2000; Saxe et al., 2008; Yap, 2004), depending on CM modalities included and the study population (Buettner et al., 2006; J. J. Mao et al., 2007; Saxe et al., 2008). While studies of the use of CM among cancer patients have been increasing, the motivation to use CM, both clinical (Gansler, Kaw, Crammer, & Smith, 2008; Velicer & Ulrich, 2008) and psychosocial (Arthur, 2012; Singh, 2005), are not fully understood. Better understanding of these reasons can increase provider awareness of CM use in general and may facilitate discussion about CM use between patients and their health care providers (Bell, 2010).

Because certain CM products may interact with conventional cancer treatments and CAM efficacy studies are limited (Hietala et al., 2011; Thomas-Schoemann et al., 2011), it is essential that CM users disclose their use (Pappas & Perlman, 2002; Richardson, Masse, Nanny, & Sanders, 2004). Several studies have reported that only 33% of adult CM users reported CM use to their healthcare provider (Kennedy, 2005; NCCAM, 2010; Robinson & McGrail, 2004). Among cancer patients, disclosure rates have been reported to be higher than the general population and range from 47% to 85%, depending on type of CM used (Chao, Wade, & Kronenberg, 2008; Saxe et al., 2008). One of the main reasons patients did not disclose their CM use or interest in CM was merely because the healthcare provider did not ask (NCCAM, 2010). Similarly, patients are more likely to disclose CM use to their healthcare provider when the provider involved the patient in his/her treatment decisions (Sleath, Callahan, DeVellis, & Sloane, 2005). Nonetheless, reasons to disclose or not to disclose CM should be further researched, because an environment that promotes two-way communication between patient and healthcare provider is essential (Ben-Arye & Frenkel, 2008).

Given the frequency of CM use and yet our limited knowledge as to why cancer patients may seek, use, and disclose CM use, we designed this study to further explore these questions. Specifically, we sought to quantify knowledge, attitudes, perceptions and practices of cancer patients at Loma Linda University Medical Center (LLUMC) towards CM by conducting a mixed-methods pilot study. The study had three phases: (1) to gather and analyze preliminary qualitative data via face-to-face administered interviews (Arthur, 2012), (2) to design an appropriate survey tool, and (3) to gather and analyze quantitative data via face-to-face administered pilot surveys. This paper will report the quantitative analysis conducted on demographic predictors of CM use, reasons to use CM, disclosure to healthcare provider data (including frequencies, reasons for or against disclosure, etc.).

## 2. Materials & Methods

### 2.1 Study Design & Eligibility

Surveys were face-to-face interview-administered and conducted by three trained graduate assistants at the Loma Linda University Medical Center (LLUMC) Cancer Center infusion room. LLUMC is a tertiary care, academic medical center that serves a racially and ethnically diverse population from the Inland Empire region of Southern California. Survey administration was triangulated (Denzin, 1970) with respect to day of the week, time of day, patient demographics, and patients’ cancer characteristics. For inclusion into the study, participants had to be receiving cancer treatment from LLUMC at the Loma Linda location during the months of June – August, 2010 and be 18 years or older. Participants were selected from a convenience sample. Triangulated data collection (Denzin, 1970) and a heterogeneous group of patients were utilized to reduce selection bias by choosing a sample population that was more representative of the cancer population attending LLUMC (Morgan, 2009; Reeves, 2010).

The study protocol was approved by the Loma Linda University Adventist Health Sciences Center Institutional Review Board. All participants gave informed written consent to participate. Seventy-five percent (75%) of patients invited to participate agreed to do so and signed written consents to participate in the survey. Medical records for all participants were reviewed.

### 2.2 Survey Instrument

The survey instrument was developed to assess complementary medicine (CM) use, disclosure, beliefs, and perceptions among cancer patients receiving conventional treatment at LLUMC and was developed from qualitative data collected (Arthur, 2012) from the same study population. Participants were asked about past and/or current use of different modalities of CM: dietary change or nutritional status, dietary supplements (including vitamins but non-herbal), green tea, herbal tea, herbs or herbal supplements (not including tea), movement-based relaxation techniques (e.g. yoga or tai chi), other relaxation techniques, meditation, prayer, spirituality (other than prayer), acupuncture/acupressure, use of a complementary and alternative medicine provider (other than an acupuncturist), or other. Examples of relaxation techniques could include guided imagery, deep breathing, music therapy, progressive muscular relaxation, massage, and cognitive-behavioral therapies. If participants expressed uncertainty about the definition or concept of “spirituality,” interviewers were prompted to state the National Cancer Institute’s definition of spirituality which is:

Having to do with deep, often religious, feelings and beliefs, including a person’s sense of peace, purpose, connection to others, and beliefs about the meaning of life. (NCI) Spirituality has also been more generally defined as the development of inner peace or the foundations of happiness.

### 2.3 Assessment of CM Use

Univariate analysis of different CM modalities was assessed using dichotomous, yes/no outcome variables. The univariate analysis included five different outcome variables: four different modalities of CM (biologically-based therapies, mind-body therapies, prayer, and use of a CAM provider) and use of any CM modality as a cancer therapy. Table 2 illustrates the items that were included in each CM modality. Use of any CM modality as a cancer therapy was a collapsed variable that was “yes” if the participant responded “yes” to using their CM with the intent to treat physical or emotional side-effects from the cancer and/or cancer treatment, prevent the cancer from recurring, help fight the cancer, or to cure the cancer (Table 2).

Four dichotomous predictor variables were assessed: gender, age (less than 60 years or greater than or equal to 60 years), ethnicity (Hispanic or non-Hispanic), and race (Caucasian or non-Caucasian). All variables were collapsed with the exception of gender. Age was a continuous variable on the survey tool and sixty years was chosen because it was the mean of the study population. Various Hispanic origin ethnicities were collapsed into Hispanic, though the majority of Hispanics were of Mexican-origin. All non-Caucasian races were collapsed since approximately 60% of the study population was Caucasian and a variety of other minority groups were sampled.

### 2.4 Data Analysis

Data entry was conducted in Microsoft Excel 2007. An alpha-level of 0.05 was used for all analyses. Sample size and power analyses were conducted using SAS Power and Sample Size 3.1. Data analysis was conducted using SAS 9.2. The Proc Freq/all command was used in the univariate analysis to determine odds ratios (OR) and significance.

## 3. Findings

### 3.1 Demographic Characteristics

Thirty-nine females (67.2%) and 19 males (32.8%) were surveyed. Thirteen percent (13.8%) of participants were Hispanic and 86.2% were non-Hispanic. While 60.3% of participants were Caucasian non-Hispanic, the remaining 39.7% were racially diverse and included African-Americans, Asians, bi-racial, and (native) American-Indian participants. Female participants’ age ranged from 34 to 88 years with a mean of 58.8 years (standard deviation, 13.2). Male participants’ age ranged from 41 to 78 with a mean of 62.2 years (standard deviation, 10.9). Overall, the study population was a mid-socioeconomic status group of participants. Seventy-seven percent (77.6%) of participants reported achieving an educational level of some college or greater and 65.5% of participants reported a household income of $50,000 or greater. Although a higher percentage of women compared to men (81.6% versus 73.7%) reported achieving some college or greater, less women reported having a household income of $50,000 or greater (46.2% versus 73.7%). Characteristics are stratified by gender in Table 1.

### 3.2 Health Factors

Of all participants, 27.5% were diagnosed with breast cancer, 15.5% were diagnosed with colon/rectal cancer, 10.3% with lung cancer, 10.3% with Non-Hodgkin Lymphoma, and 8.6% with prostate cancer. Twenty-seven percent (27.6%) of participants had experienced recurrence and the mean time since diagnosis was 3.26 years. Most participants (84.5%) had or were receiving combination therapy (two or more conventional therapies, such as chemotherapy, radiation, surgery), whereas 15.5% of participants were receiving chemotherapy exclusively. Characteristics are stratified by gender in Table 1.

### 3.3 Complementary Medicine (CM) Use

Of 58 participants, there were 57 (98%) CM users, including dietary supplements, and only one non-user (female) who responded “I don’t use or do anything outside of what my physician/health care provider prescribes.” There were 51 participants (87.9%) who used CM specifically as a cancer treatment.

#### 3.3.1 Prevalence

Among all female participants, prayer was the most common CM modality used (94.9%), followed by biologically-based therapies (92.3%), specifically, dietary change (87.2%) and dietary supplements (79.5%). Among all male participants, biologically-based therapies were the most common CM modality used; specifically, dietary change (79.0%) and dietary supplements (79.0%), followed by prayer (68.4%). Use of a CAM provider was the least common CM modality used among both females and males (12.8% & 10.5%, respectively). Five percent (5.3%) of females and 10.5% of males used prayer exclusively as their CM, and therefore used no other CM modality. Complementary medicine (CM) use is stratified by gender in Table 2.

#### 3.3.2 Reasons to Use

Eighty-nine percent (89.7%) of all females and 84.2% of all males reported using complementary medicine, of any modality, as a cancer therapy. Among females and males, the most common reason to use CM as a cancer therapy was “to help fight the cancer” (84.6% & 79.0%, respectively), followed by “to prevent recurrence” (76.3% & 63.2%, respectively). The least common reason to use CM as a cancer therapy, reported by females, was “to cure the cancer” (69.2%), whereas men least reported that they used CM “to relieve their emotional symptoms” (47.2%). Refer to Table 2.

### 3.4 Demographic Predictors of CM Use

#### 3.4.1 Gender

Females were eight times more likely (OR = 8.5) to use prayer as CM compared to males. Only two females reported they did not use prayer as CM. However, females were not more likely to use other CM modalities and were not more likely than men to use any type of CM as a cancer therapy.

#### 3.4.2 Age

Participants less than 60 years of age were 2.5 times more likely to report the use of prayer as CM compared to older participants (OR = 2.556), yet were less likely to report the use of biologically-based CM (OR = 0.473) and mind-body therapies (OR = 0.333). These point estimates indicate that participants 60 years of age or older were approximately twice as likely to report the use of biologically-based CM and three times as likely to report the use of mind-body therapies. Point estimates greater than or equal to two are noteworthy, though none were statistically significant.

#### 3.4.3 Ethnicity

Hispanic participants were 3 times more likely to report use of mind-body therapies (OR = 3.18). An odds ratio for use of any CM as a cancer therapy could not be computed, because all Hispanic participants reported to use CM as a cancer therapy.

#### 3.4.4 Race

Caucasians were three times more likely to report the use of biologically-based CM (OR = 3.00) compared to non-Caucasians. An odds ratio for use of prayer and use of any CM as a cancer therapy could not be computed because all non-Caucasians used prayer and any CM as a cancer therapy. An odds ratio for use of a CAM provider could not be computed because zero non-Caucasians reported to use a CAM provider (Table 3). Each predictor in the univariate model had greater than 90% power.

### 3.5 Disclosure of CM Use

#### 3.5.1 Prevalence

Forty participants were asked questions regarding disclosure of CM use. The majority of participants reported to have disclosed their CM use to a health care provider, and proportionally more women than men had disclosed (72.0% & 53.3%, respectively). Of those who reported to have disclosed their CM use, disclosure to an oncologist was the most common (66.7% among females & 50.0% among males), followed by disclosure to a primary care physician (38.9% among females & 37.5% among males) and disclosure to a nurse (22.2% among females & 37.5% among males). Over one third of females (38.9%) and 12.5% of males reported to have disclosed their CM use to more than one health care provider.

Those who reported to not have disclosed their CM use to a health care provider were asked who they were most likely to tell. Most responded they would disclose to family and/or friends (28.6% among females & 42.9% among males), a primary care physician (28.6% among females & 28.6% among males), or an oncologist (28.6% among females & 14.3% among males). Less men than women reported they would disclose to a physician (Table 4).

#### 3.5.2 Reasons to Disclose

Participants (n = 40) were asked to pick the statement that *best* completed this sentence: “Sometimes I do not ask or tell my oncologist about my CAM use because….” Out of seven formatted responses and “other,” which allowed for an open-ended response, the most common response was because “he/she is most concerned about my cancer and cancer treatment” (27.5%) and “other” (27.5%), followed by “I just did not think to tell him/her” (22.5%). The most common reason participants (n = 11) reported to have selected “other” was because they tell their providers (72.7%). Other open-ended responses were “I’m comfortable speaking with my physician(s)” (18.2%) and “he told me to take these things [CAM]” (9.1%). Refer to Table 5.

## 4. Discussion

Our findings indicate a very high prevalence of complementary medicine (CM) use among cancer patients receiving conventional treatment at Loma Linda University Medical Center (LLUMC). Although the prevalences found for use of different CM modalities were wide-ranging (10% to 95%), an 88% prevalence for any type of CM use as a cancer therapy was higher than other estimates, which typically range from 40% to 83% (Boon et al., 2000; Buettner et al., 2006; Cassileth et al., 1984; DiGianni et al., 2002; Ernst & Cassileth, 1998; Ganz et al., 2002; Greenlee et al., 2009; Habermann et al., 2009; Henderson & Donatelle, 2004; Lawsin et al., 2007; Lee et al., 2000; Lo et al., 2009; Mao et al., 2007; Mao, 2011; Morris et al., 2000; Richardson et al., 2000; Saxe et al., 2008; Yap, 2004). The reported disclosure proportions in this study (72% among women and 53% among men) were higher than the general population’s estimate for CM disclosure, but comparable to other reported disclosure proportions among patients with a serious illness (47% to 85%) (Chao et al., 2008; Saxe et al., 2008). Our main findings are (1) differentiating CM modalities shows that different prevalences exist for each modality, (2) differentiating CM modalities shows that different predictors of CM use exist for each modality, (3) reasons to use CM vary by gender, and (4) disclosure proportions also vary by gender.

### 4.1 CM Use

While overall CM use was more prevalent among women as found in other studies (Barraco et al., 2005; F. L. Bishop & Lewith, 2010; Lawsin et al., 2007), trends of most prevalent CM modalities found in this study were similar among women and men. For instance, biologically-based CM therapies were most popular among both women (92.3%) and men (89.5%) and use of a CAM provider was least popular (10%) (Gansler et al., 2008; J. Mao et al., 2008; Richardson et al., 2004; Upchurch, 2007; Wade, Chao, Kronenberg, Cushman, & Kalmuss, 2008). Within types of biologically-based therapies, dietary change and dietary supplements were most prevalent, followed by green tea, herbal tea, and herbs or herbal supplements for both genders. Similarly, trends for the different types of mind-body therapies were comparable among both genders; prayer and spirituality were most popular and movement-based relaxation was least utilized as CM. These results are in line with other studies that find a high use of prayer as CM (Bishop & Lewith, 2010; Gansler et al., 2008; Lawsin et al., 2007; J. J. Mao et al., 2007; Richardson et al., 2004; Richardson et al., 2000) and a lower utilization of a CAM provider (Gansler et al., 2008; J. Mao et al., 2008; Richardson et al., 2004). In addition, women were eight times more likely to use prayer as CM for general health compared to men, as supported by other studies (Gansler et al., 2008; Sleath et al., 2005).

Our unadjusted univariate analyses differed from other demographic studies that found that Caucasian, younger female cancer patients of higher education were more likely to utilize CM (Bishop & Lewith, 2008; Bishop & Lewith, 2010; Lawsin et al., 2007). Our study demonstrated that CM use, specifically as a cancer therapy, did not significantly vary by gender, age, or education. Other studies that reported CM usage from under-represented populations have found that CM use was high among poor, older, and ethnic minority adults (Goldstein et al., 2005). This inclusivity of results may contribute to our null findings when analyzing the overall use of CM among a heterogeneous group of patients. Furthermore, it indicates the importance of differentiating CM modalities when assessing CM use among cancer patients.

Associations between race, ethnicity and CM use remain uncertain. Approximately 38 studies have shown that minority groups use CM less than Caucasians, whereas at least 15 studies have found that minority groups use CM more (Bishop & Lewith, 2008; Goldstein et al., 2005). Divergent findings may in part be due to underrepresented populations, for example, minority groups accounting for only approximately 10% of a sample population (Gansler et al., 2008; Goldstein et al., 2005), and types of CM included in a study (Hsiao et al., 2006). Our findings do not resolve this uncertainty. All Hispanic and non-Caucasian participants used at least one CM modality as a cancer therapy, but none of them used a CAM provider.

The reasons to use CM varied by gender. More than two-thirds of the women responded ‘yes’ to each of the reasons listed on the survey, suggesting women sought and used CM as a medical pluralistic approach to help them better achieve holistic well-being (Belliard, 2005; NCCAM, 2010; Richardson et al., 2004). Other reasons for increased use among women, reported by past studies, include a greater sense of control of self, a more personal focus on health, and an overall increased use of health/medical services (Brett, 2001; Gansler et al., 2008).

### 4.2 Disclosure of CM Use

Our findings are consistent with prior studies that too have reported a higher disclosure rate among patients with a life-threatening illness compared to the general adult population (Goldstein et al., 2005; Richardson et al., 2004; Saxe et al., 2008; Sleath et al., 2005). Our findings are also consistent with prior data that women are more likely to disclose their CM use to a health care provider than men (Harrigan, 2011; NCCAM, 2010; Richardson et al., 2004). Women were also more likely to tell more than one health care provider. Reasons for this higher disclosure rate among women may be because women tend to see doctors more often (Brett, 2001), ask more questions, and have longer encounters with physicians compared to men (Falik, 1996; Richardson et al., 2004). All respondents were more likely to disclose CM use to a physician (NCCAM, 2010) rather than another healthcare provider.

The most common reason cited for a patient’s non-disclosure of his/her CM use to the oncologist was because the patient perceived the role of the oncologist to be most concerned with cancer and cancer treatment. While there is no perfectly comparable research, findings reported by NCCAM (2010 & 2006) stated that the number one reported reason for a patient’s non-disclosure was that the health care provider never asked the patient, followed by the response that the patient didn’t know he/she should disclose (NCCAM, 2010). In addition, a higher proportion of men reported to disclose their CAM use to a friend or relative as opposed to a health care provider. Among the general population, non-disclosure to a health care provider among men may be related to the lower utilization of health care compared to women (Bonhomme, 2007; Brett, 2001; Brott et al., 2011), however, male cancer patients would see their physicians as much as women. It may simply be because women more often take an active role in their health care compared to men (Bonhomme, 2007; Brett, 2001). Nevertheless, there is a lack of literature on reasons for or against disclosure of CAM use.

### 4.3 Strengths & Limitations

This study has a number of limitations. As this was a non-randomized, small, convenience sample, there is potential for systematic bias. However, selection methods were designed to reduce selection bias by sampling over a period of time, on every day of the week, and at various times during the day, and were inclusive of all patient types. While investigator/interviewer biases are possible, interviewers were trained to follow protocol to recruit participants and a standardized method to survey administration was used. The small sample sizes after stratification made it difficult to compute an accurate and fully-adjusted model of demographic predictors of CM use. However, in a univariate model, statistical power exceeded 90% for each predictor.

Despite these limitations, our study provides a variety of insights on CM use among cancer patients. The inclusion of minorities and their high prevalence of CM use sheds light on the practice of medical pluralism (Belliard, 2005). Differentiating CM modalities as separate outcome variables not only adjusts for confounding by CM modality, but illustrates that predictor variables among the CM modalities are different. Because this is a cancer population, making a distinction between CM modalities that can interact with conventional treatment, such as herbs or antioxidants interacting with chemotherapy, is vital (Hietala et al., 2011; Thomas-Schoemann et al., 2011). Selection bias was reduced do to recruiting face-to-face which gave us a very high response rate. Lastly, the survey tool used and survey administration were a part of a mix-methods study design to limit information bias and improve validity of the data.

### 4.4 Conclusion

Future research should repeat this present study in a community hospital setting to increase sample size, increase the range of patient characteristics, and to be more inclusive of minorities and low SES patients. Further investigation is required to better determine an association between race, ethnicity, age, sex, SES and CAM use among cancer patients. CAM use should be differentiated by CAM modalities, as it is likely that lower SES cancer patients will not be financially able to seek expensive CAM modalities, such as a CAM provider (Richardson et al., 2004). Because possible interactions exist with certain cancer treatments, these findings of high CM use among patients undergoing such treatments iterates the importance of CAM efficacy studies.

The high utilization of CAM among cancer patients and non-disclosure proportions suggests the implementation of integrative medicine may improve physician-patient relationships, influence health care providers to provide patients with the risks and benefits of CAM use, which may improve survivor quality of life, and may help prioritize research investigating reasons to use CAM and efficacy and safety of CAM use.

## Figures and Tables

**Table 1:** Characteristics of Study Population

Characteristic	Females n = 39	Males n = 19
Age (years)	(%)	(%)
18–34	5.1	0.0
35–49	20.5	15.8
50–59	28.2	5.3
60–69	25.6	47.4
≥ 70	20.5	31.6
Ethnicity		
Hispanic	15.8	11.1
non-Hispanic	84.2	88.9
Race		
Caucasian, non-Hispanic	61.5	57.9
African-American, non-Hispanic	7.7	5.3
Chinese, non-Hispanic	0.0	5.3
Filipino, non-Hispanic	5.1	0.0
Other Asian, non-Hispanic	0.0	5.3
Bi-racial, non-Hispanic	5.1	0.0
American-Indian, non-Hispanic	2.6	10.5
Other	17.9	13.3
Education		
High school or less	18.4	26.3
Some college or greater	81.6	73.7
Household income		
$50,000 or less	53.9	26.3
≥ $50,000	46.2	73.7
Cancer Diagnosis		
Breast	41.0	0.0
Colon/rectal	15.4	15.8
Leukemia	0.0	5.3
Lung	10.3	10.5
Melanoma	5.1	5.3
Non-Hodgkin Lymphoma	10.3	10.5
Ovarian	10.3	0.0
Prostate	0.0	26.3
Other	7.7	26.3
Experienced recurrence		
Yes	34.2	15.8
No	65.8	84.2
Conventional Treatment		
Chemotherapy, exclusively	15.4	15.8
Combination therapy	84.6	84.2

**Table 2:** Prevalence (%) of and reasons to use complementary medicine

Complementary Medicine Use	Females (%)* n = 39	Males (%)* n = 19
Biologically-based therapies	92.3	89.5
Dietary Change	87.2	79.0
Dietary supplements**	79.5	79.0
Green Tea	56.4	31.6
Herbal Tea	46.2	21.1
Herbs or herbal supplements	25.6	15.8
Mind-body therapies	71.8	73.7
Movement-based relaxation	18.0	15.8
Other relaxation	43.6	52.6
Meditation	38.5	26.3
Spirituality	51.3	52.6
Prayer	94.9	68.4
Prayer (exclusively)	5.3	10.5
CAM provider	10.3	10.5
CAM as a cancer therapy#	89.7	84.2
To relieve physical symptoms	71.8	57.9
To relieve emotional symptoms	74.4	47.4
To prevent recurrence	76.3	63.2
To help fight the cancer	84.6	79.0
To cure the cancer	69.2	57.9

* percentage of all female and male participants, respectively

** vitamin, animal-origin, or micro-organism supplements

# “yes” to any of the reasons below

**Table 3:** Odds ratios to determine predictors of CM use – univariate analysis

Predictor	Biologically-based	Mind-Body Therapies	Prayer	CAM Provider	Use of CM as a cancer therapy
Female	1.41	0.91	8.54*	0.97	1.64
Male	1.00	1.00	1.00	1.00	1.00
18 – 60 years	0.47	0.33	2.56	0.63	1.01
≥ 60 years	1.00	1.00	1.00	1.00	1.00
Hispanic	0.64	3.18	1.00	1.14*	#
non-Hispanic	1.00	1.00	1.00	1.00	1.00
Caucasian**	3.00	0.76	#	#	#
Non-Caucasian	1.00	1.00	1.00	1.00	1.00

* Statistically significant value (p-value < 0.05)

# Cell contains zero, thus cannot compute OR

** Regardless of ethnicity (there was one female Caucasian, Hispanic)

**Table 4:** Disclosure of complementary medicine use to a health care provider

Did you disclose your complementary medicine use to a health care provider?	Females (%) n = 25*	Males (%) n = 15*
Yes	72.0	53.3
No	28.0	46.7
*If yes, to whom?*#	n = 18	n = 8
Oncologist	66.7	50.0
Nurses	22.2	37.5
Primary care physician**	38.9	37.5
Dietician/Nutritionist	16.7	25.0
Radiologist	5.6	37.5
PA	0.0	6.7
More than 1	38.9	12.5
*If no, who are you most likely to tell?*#	n = 7	n = 7
Family and/or friends	28.6	42.9
Primary care physician**	28.6	28.6
Oncologist	28.6	14.3
Insurance provider	14.3	14.3
No one	14.3	0
I don’t know	0	14.3
More than 1	0	14.3

* This question was not on the first 18 surveys administered. Female who did not currently use any CAM excluded.

# Open-ended question.

** Other terms used were “general practitioner,” “family physician,” or “physician.”

**Table 5:** Reasons to disclose or not to disclose use of complementary medicine to a health care provider

Pick the statement that best completes this sentence: Sometimes I do not ask or tell my oncologist about my CAM use because,*	(%) n = 40
He/she is most concerned about my physical well-being	10.0
He/she is most concerned about my cancer and cancer treatment	27.5
I just did not think to tell him/her	22.5
I feel more comfortable talking to another health care provider.	2.5
I feel that I will be judged.	2.5
I feel that my oncologist will view me negatively.	2.5
“I don’t know”	5.0
Other**	27.5
“I tell my providers”	72.7
“I’m comfortable speaking with physician”	18.2
“He told me to take these things [CAM]”	9.1

* This question was not on the first 18 surveys administered.

** “Other” allowed for an open-ended response.
